# Mental health in young adults born extremely preterm or extremely low birthweight with contemporary neonatal intensive care

**DOI:** 10.1017/S0033291722002276

**Published:** 2023-08

**Authors:** Alice C. Burnett, Rheanna M. Mainzer, Lex W. Doyle, Katherine J. Lee, Peter J. Anderson, Diana Zannino, Julianne Duff, George C. Patton, Jeanie L. Y. Cheong, for the Victorian Infant Collaborative Study Group

**Affiliations:** 1 Victorian Infant Brain Studies, Murdoch Children's Research Institute, Melbourne, Australia; 2 Premature Infant Follow-Up Program, Royal Women's Hospital, Melbourne, Australia; 3 Neonatal Medicine, Royal Children's Hospital, Melbourne, Australia; 4 Department of Paediatrics, University of Melbourne, Melbourne, Australia; 5 Clinical Epidemiology and Biostatistics Unit, Murdoch Children's Research Institute, Melbourne, Australia; 6 Department of Obstetrics and Gynaecology, University of Melbourne, Melbourne, Australia; 7 Turner Institute for Brain & Mental Health, School of Psychological Sciences, Monash University, Melbourne, Australia; 8 Mercy Hospital for Women, Heidelberg, Victoria, Australia; 9 Centre for Adolescent Health, Murdoch Children's Research Institute, Melbourne, Australia

**Keywords:** ADHD, adult, anxiety, depression, extremely preterm, mental health

## Abstract

**Background:**

For infants born in the contemporary era of neonatal care, little is known about adult mental health outcomes of extremely preterm birth (EP; <28 weeks' gestation) or extremely low birthweight (ELBW; <1000 g). This study aimed to compare attention deficit hyperactivity disorder (ADHD), anxiety, mood, and substance use disorder prevalence in young adults born EP/ELBW and normal birthweight (NBW; >2499 g) controls, and to compare change in prevalence of mental health symptoms and disorders from 18 to 25 years.

**Methods:**

Participants were a prospective geographical cohort of 297 consecutive survivors born EP/ELBW during 1991–1992 and 260 NBW controls. At age 25 years, 174 EP/ELBW and 139 NBW participants completed the Adult ADHD Rating Scale, Structured Clinical Interview for DSM-IV Disorders, Beck Anxiety Inventory, and Center for Epidemiologic Studies Depression Scale-Revised. Data from follow-up at 18 years were also utilized. Multiple imputation was used to account for attrition.

**Results:**

Mental health outcomes at 25 years were similar between groups: prevalence rates were ADHD 7% *v.* 5%; anxiety 32% *v.* 27%; mood 38% *v.* 35%; substance use 12% *v.* 14% in the EP/ELBW and NBW groups, respectively. In both groups, ADHD declined between 18 and 25 years [odds ratio (OR) per year = 0.87, 95% confidence interval (CI) 0.79–0.95], and generalized anxiety disorder and major depressive episode became more common (OR 1.22, 95% CI 1.10–1.35 per year; OR 1.20, 95% CI 1.10–1.30 respectively).

**Conclusions:**

This contemporary EP/ELBW cohort has comparable young adult mental health outcomes to controls, and similar patterns of change in mental health from late adolescence.

## Introduction

Extremely preterm birth (EP; birth <28 weeks' gestation) and extremely low birthweight (ELBW; birthweight <1000 g) are risk factors for behavioral and emotional difficulties in childhood, particularly attention deficit hyperactivity disorder (ADHD) and anxiety (Johnson & Marlow, 2014). Many mental health disorders emerge in adolescence and symptoms may evolve during the transition to adulthood (Caspi et al., 2020). Adults born EP/ELBW may be more vulnerable to mental health problems compared with those born normal birthweight (NBW; >2499 g) (Anderson et al., 2021; Mathewson et al., 2017; Nosarti et al., 2012). However, studies to date are from adult EP/ELBW cohorts with low survival rates. EP/ELBW infants' survival more than doubled in the 1990s compared with pre-1990, with survival particularly increasing for the smallest and most immature infants (Doyle & Victorian Infant Collaborative Study Group, 2004). As the earliest surviving infants born EP/ELBW in the contemporary era of neonatal care are only now reaching adulthood, it has become possible to ascertain whether they are at increased risk of adult mental health difficulties compared with their NBW peers, and whether this picture changes from adolescence to young adulthood.

Aspects of adult mental health have been examined in studies of people born EP/ELBW or very preterm/very low birthweight (VP; <32 weeks' gestation/VLBW; <1500 g) before the 1990s. Individual participant data (IPD) meta-analysis indicates a strong association between VP/VLBW birth and ADHD, with around a fivefold increase in risk compared with controls (Anderson et al., 2021). VP/VLBW cohort studies have not conclusively found an increased prevalence of anxiety and mood disorders in the adult period compared with their NBW peers (Lærum et al., 2017; Mathewson et al., 2017; Robinson et al., 2020), but an IPD meta-analysis indicates a marginal increase in the odds of these disorders in VP/VLBW adults compared with controls, suggesting cohort studies may be underpowered to detect small effects in this domain (Anderson et al., 2021). While adults born VP/VLBW may face increased risk in some mental health domains, their rates of alcohol and substance use disorders (SUD) may be lower than controls' (Strang-Karlsson et al., 2008; Van Lieshout, Boyle, Saigal, Morrison, & Schmidt, 2015). However, it is possible that mental health outcomes for adults born EP/ELBW in the contemporary era may differ to the findings outlined above for two main reasons. Firstly, EP/ELBW birth confers an increased biological vulnerability compared with VP/VLBW birth, and secondly, as noted above, the profile of survivorship for babies born EP/ELBW changed dramatically in the contemporary era of neonatal care. Thus, it remains to be determined whether those born EP/ELBW in the contemporary era experience similar adult outcomes to those born in earlier eras.

There are limited longitudinal data examining mental health in EP/ELBW cohorts from adolescence to adulthood. In one of the earliest geographical cohorts of the contemporary era, we found elevated ADHD but similar anxiety, mood, and SUD prevalence in older adolescents born EP/ELBW in 1991–1992 compared with NBW controls at 18 years (Burnett et al., 2014). Longitudinal evidence from pre-1990 cohorts indicates rates of ADHD decrease from adolescence to adulthood, although the risk remains elevated for VP/VLBW adults (risk ratio 3.29, 95% confidence interval 1.39–7.81) (Breeman, Jaekel, Baumann, Bartmann, & Wolke, 2016). Evidence is mixed as to whether the trajectory of internalizing symptoms (i.e. anxiety and/or depression symptoms) from adolescence into adulthood differs between ELBW and NBW young people (Bachmann, Risnes, Bjørngaard, Schei, & Pape, 2021; Van Lieshout et al., 2018; cf. Jaekel, Baumann, Bartmann, & Wolke, 2018; Lærum et al., 2017). Evidence on mental health outcomes for contemporary EP/ELBW individuals is needed in order to provide up-to-date guidance for mental health surveillance and service provision.

We aimed to compare the prevalence of ADHD, anxiety, and mood symptoms and disorders, and SUD in young adults born EP/ELBW in 1991–1992 with NBW controls at 25 years. Based on our findings in the same cohort in adolescence, we hypothesized that young people born EP/ELBW would have higher rates of ADHD, but similar anxiety, depression, and substance use outcomes compared with NBW controls. Our second aim was to determine whether the prevalence of symptoms and diagnoses of key mental health conditions changed in each group from late adolescence to adulthood, and whether this varied by birth group. We expected that ADHD symptoms and prevalence would decline with age in both groups but do so more slowly in the EP/ELBW group than the NBW group, while current anxiety, mood, and SUDs would increase similarly with age in both groups.

## Methods

### Participants

Participants were part of a prospective geographical cohort study, comprising all 297 surviving infants born EP/ELBW in the state of Victoria, Australia, during 1991 and 1992. A control group of 260 surviving NBW infants was recruited contemporaneously at birth and matched to the EP/ELBW group for sex, expected due date, maternal health insurance status (private/none), and maternal country of birth (primarily English-speaking/not). Participants included 31 families where two twins were both enrolled in the study [28 families (56 participants) in the EP/ELBW group, and 3 families (6 participants) in the NBW group]. The cohorts have been previously assessed at 2, 5, 8, and 18 years of age (Anderson & Doyle, 2003; Burnett et al., 2014; Doyle, 2001; Victorian Infant Collaborative Study Group, 1997).

### Measures and procedure

At 25 years of age, participants were reassessed for a study of young adult health outcomes (Cheong et al., 2019). Current and previous study waves were approved by the Human Research Ethics Committees at the Royal Women's Hospital, Mercy Hospital for Women, Monash Medical Centre, and the Royal Children's Hospital. Participants' parents consented to the original study and follow-up waves during childhood, and participants themselves gave informed consent to participate in the young adult phase. Medical data collected in the newborn period included gestational age, birthweight, birthweight standard deviation score (BW-SDS), major brain injury (grade III/IV intraventricular hemorrhage or cystic periventricular leukomalacia), postnatal corticosteroid treatment, and neonatal surgery. Demographic and developmental information was recorded, including maternal education (lower: <12 years of schooling; higher: 12+ years), the presence of major neurosensory disability [any of blindness, deafness, moderate/severe cerebral palsy, and IQ more than 2 standard deviations (s.d.) below the control group mean], and IQ at age 8 and 18 years. Adolescent mental health outcomes were collected at 18 years (Burnett et al., 2014), using the ADHD module of the Children's Interview for Psychiatric Syndromes (ChIPS) (Weller, Weller, Rooney, & Fristad, 1999), the Beck Anxiety Inventory (BAI) (Beck & Steer, 1990), the Center for Epidemiologic Studies Depression Scale-Revised (CESD-R) (Eaton, Muntaner, Smith, Tien, & Ybarra, 2004), and the Structured Clinical Interview for DSM-IV (SCID-IV) (First, Spitzer, Gibbon, & Williams, 2002).

At 25 years, mental health was assessed via questionnaires and clinical interviews conducted by research assistants who had Masters-level training in clinical psychology and had provisional or full registration as psychologists. Assessors were blinded to birth group and previous data. Current ADHD symptomatology was captured using the Adult ADHD Self-Rating Scale (Kessler et al., 2005). Participants reporting symptoms meeting the Part A threshold of this scale were considered to show increased ADHD symptoms and a follow-up interview was used to determine whether the other diagnostic criteria were met. Current anxiety and depression symptoms were again reported using the BAI and CESD-R. In cases of missing data, scores on these questionnaires were prorated where a sufficiently small number of items were missing as per the manuals (Beck & Steer, 1990; Eaton et al., 2004). The total scores of the BAI and CESD-R were highly positively skewed, making the mean difficult to interpret, and the distributions of these scores were similar between birth groups (online Supplementary Table S1). In light of these factors, and to ensure comparability with the report of these outcomes at 18 years (Burnett et al., 2014), the total scores were dichotomized at the standard clinical cut-off for further analysis (Beck & Steer, 1990; Eaton et al., 2004), with scores of 16+ considered to reflect elevated symptoms for both scales. Composite outcomes reflecting the presence of either high BAI or CESD-R scores at each timepoint were also examined to address heterotypic continuity amongst internalizing symptoms. The SCID-IV was used to identify past or current symptoms meeting criteria for a mental health diagnosis in the domains of anxiety, mood, and substance use. DSM-IV disorders were assessed to ensure comparability with 18-year data. Specific disorders assessed are listed in online Supplementary Table S2.

### Analysis

Odds ratios (ORs) were estimated to compare 25-year outcomes in the EP/ELBW and NBW groups (aim 1) using logistic regression models, fitted using generalized estimating equations (GEE) with exchangeable correlation structure, and are reported with robust (sandwich) standard errors to account for clustering of multiple births (i.e. twins) within families. This approach takes into account the correlation (non-independence) between the siblings (Carlin, Gurrin, Sterne, Morley, & Dwyer, 2005). Analyses for aim 1 were conducted using multiple imputation by chained equations to minimize the impact of missing data, with imputation performed separately for each outcome and by EP/ELBW or NBW group (except for ADHD diagnosis, where the groups were combined due to low cell counts). Imputation models included the outcome, covariates included in the sensitivity analysis (listed below), and additional auxiliary variables from the 8- and 18-year follow-ups that were correlated with the outcome (see online Supplementary Table S3). Variables were imputed using either logistic or linear regression models for binary and continuous variables respectively, with a total of 50 imputations produced and final OR estimates obtained using Rubin's rules (Rubin, 1987).

In order to examine the group-level change in the odds of mental health symptomatology between 18 and 25 years (aim 2), we estimated the OR of experiencing current mental health symptoms per 1-year increase in age for ADHD outcomes, high BAI and CESD-R scores, the prevalence of generalized anxiety disorder (GAD), current major depressive episode (MDE), and current SUD (any of alcohol or non-alcohol dependence or abuse). ORs were estimated using logistic mixed-effects models applied to the 18- and 25-year data with a random intercept to account for correlation among repeated measures, or GEEs with an exchangeable correlation structure and robust standard errors to allow for repeated observations within individuals if mixed models failed to converge. A group-by-age interaction term was used to examine differential effects of time in the two groups and the interaction term was dropped from the model if the evidence for an interaction was weak (*p* > 0.05). Multiple imputation was not performed when analyzing changes in mental health difficulties over time as mixed-effects models use all available outcome data, including individuals with a measurement at just one timepoint. Sensitivity analysis was conducted for both aims. In the sensitivity analysis, models were adjusted for sex (due to its associations with mental health), BW-SDS (as a marker of intrauterine growth relative to gestational age), and lower maternal education (as a proxy for socioeconomic status), and excluded participants with low IQ scores (to address any impact of intellectual difficulties on results). Low IQ was defined as <−2 s.d. from the control mean at 18 years (or at 8 years if 18-year data were missing). Supplementary analyses report estimates from complete case analyses, including a descriptive examination of intra-individual stability. Analyses were conducted using Stata 16 (StataCorp, 2019).

## Results

### Sample characteristics and loss to follow-up

At 25 years, 59% of the EP/ELBW cohort and 53% of the NBW cohort had available mental health outcome data (Table 1). The two groups were similar in age [EP/ELBW: 25.4 years (s.d. 0.7); NBW: 25.3 years (s.d. 0.9)]. The EP/ELBW participants were broadly representative of the original cohort in their perinatal characteristics, although those with major neonatal brain injury were less likely to have data at 25 years (Table 1). In both birth groups, females were more likely to participate than males, as were those with higher IQ at 18 years. In the EP/ELBW group, those who participated at 25 years had similar or slightly higher rates of psychopathology at 18 years compared with non-participants. In the control group, however, the reverse was true, with those who participated at 25 years having better mental health in adolescence compared with non-participants.

**Table 1.** Characteristics of extremely preterm/extremely low birthweight (EP/ELBW) and normal birthweight (NBW) control participants assessed and not assessed at 25 years

	EP/ELBW		NBW controls	
Perinatal	Assessed *n* = 174	Not assessed *n* = 123	Assessed *n* = 139	Not assessed *n* = 121
Gestational age (weeks) *M* (s.d.)	26.6 (2.0)	26.8 (1.8)	39.2 (1.5)	39.2 (1.4)
Birthweight (g) *M* (s.d.)	884 (157)	894 (167)	3375 (463)	3400 (408)
Birthweight s.d.-score *M* (s.d.)	−0.34 (1.13)	−0.39 (1.16)	0.14 (0.94)	0.15 (0.89)
Maternal age at birth (y) *M* (s.d.)	28.8 (5.8)	28.2 (5.8)	29.9 (4.8)	28.4 (5.6)
Female	97 (56%)	63 (51%)	89 (59%)	53 (44%)
Singleton birth	117 (67%)^a^	90 (73%)	135 (97%)^b^	117 (97%)
Small for gestational age	28 (16%)	18 (15%)	1 (1%)	0 (0%)
Major neonatal brain injury	14 (8%)	18 (15%)	0 (0%)	0 (0%)
Postnatal corticosteroids	58 (33%)	39 (32%)	0 (0%)	0 (0%)
Neonatal surgery	44 (25%)	33 (27%)	0 (0%)	0 (0%)
Age 8 years				
Maternal education ⩾12 years	81/168 (48%)	47/100 (47%)	96/135 (71%)	40/84 (48%)
Major neurosensory disability	17/171 (10%)	35/120 (29%)	1/138 (1%)	9/104 (9%)
Full-scale IQ *M* (s.d.)	98 (16)	92 (15)	108 (11)	100 (16)
Age 18 years				
Full-scale IQ *M* (s.d.)	97 (16)	91 (15)	109 (13)	100 (14)
ADHD symptoms	36/152 (24%)	6/54 (11%)	8/114 (7%)	7/38 (18%)
ADHD diagnosis	26/152 (17%)	4/55 (7%)	4/116 (3%)	7/38 (18%)
Current anxiety symptoms^c^	28/146 (19%)	6/48 (13%)	16/113 (14%)	4/33 (12%)
Current depression symptoms^d^	34/144 (24%)	9/47 (19%)	21/113 (19%)	11/35 (31%)
Any lifetime anxiety disorder	22/153 (14%)	1/55 (2%)	11/116 (9%)	5/39 (13%)
Any lifetime mood disorder	26/153 (17%)	7/55 (13%)	13/116 (11%)	10/39 (26%)
Any lifetime substance use disorder	8/153 (5%)	1/54 (2%)	3/116 (3%)	2/38 (5%)

Data are *n* (%), unless stated otherwise. Major neonatal brain injury: grade III/IV intraventricular hemorrhage/cystic periventricular leukomalacia; M, mean; s.d., standard deviation; small for gestational age: birthweight more than 2s.d. below the mean for gestational age and sex.

a Includes *n* = 17 pairs of twins (34 participants).

b Includes *n* = 1 pair of twins (2 participants).

c BAI, Beck Anxiety Inventory score in moderate/severe range (16+).

^d^ Center for Epidemiologic Studies Depression Scale Revised score in elevated range (16+).

### Mental health at 25 years in young people born EP/ELBW compared with NBW

At 25 years of age, there were only minimal group differences in any outcomes of interest (Table 2). The point estimates of group differences were consistent with a small effect of birth group but the confidence intervals were wide. Adjustment for BW-SDS, sex, and maternal education, and excluding participants with low IQ scores did not appreciably change the results. A similar pattern was observed in the complete case analysis (online Supplementary Table S4).

**Table 2.** Comparison of mental health outcomes at 25 years between extremely preterm/extremely low birthweight (EP/ELBW) and normal birthweight (NBW) control groups

	EP/ELBW proportion (95% CI)	NBW proportion (95% CI)	Odds ratio (95% CI)	*p*	Adjusted odds ratio (95% CI)	*p*
ADHD symptoms	19% (14–25%)	12% (6–18%)	1.71 (0.88–3.32)	0.11	1.77 (0.82–3.83)	0.15
ADHD diagnosis	7% (2–12%)	5% (1–9%)	1.34 (0.44–4.09)	0.60	1.74 (0.48–6.31)^a^	0.40^a^
Current anxiety symptoms^b^	27% (21–33%)	25% (18–32%)	1.10 (0.67–1.81)	0.70	1.09 (0.56–2.13)	0.79
Current depression symptoms^c^	31% (23–38%)	32% (24–40%)	0.94 (0.58–1.54)	0.82	0.83 (0.44–1.58)	0.58
Current anxiety or depression symptoms^b^^,^^c^	51% (44–58%)	46% (38–55%)	1.20 (0.79–1.82)	0.40	1.12 (0.66–1.92)	0.68
Any lifetime anxiety disorder	32% (25–39%)	27% (19–35%)	1.31 (0.77–2.21)	0.32	1.50 (0.82–2.76)	0.19
Any lifetime mood disorder	38% (31–45%)	35% (26–44%)	1.12 (0.69–1.82)	0.65	1.01 (0.55–1.87)	0.98
Any lifetime substance-use disorder	12% (7–16%)	14% (8–21%)	0.80 (0.39–1.67)	0.56	0.84 (0.34–2.12)^a^	0.72^a^

a Results are from logit regression due to failure of GEE models to converge.

b On Beck Anxiety Inventory.

c On Center for Epidemiologic Studies Depression Scale-Revised. Estimates are obtained using multiple imputation. Adjusted results are adjusted for sex, birthweight s.d. score, lower maternal education, and exclude those with IQs more than 2s.d. below the control group mean. CI, confidence interval.

### Changes in mental health outcomes from 18 to 25 years

The effects of increasing age on the odds of current mental health difficulties were similar between the EP/ELBW and NBW groups (Table 3). The odds of ADHD symptoms remained stable over time but the odds of participants meeting diagnostic criteria for ADHD reduced over time. There was some evidence of a slight increase over time in the odds of questionnaire-reported anxiety symptoms, which weakened after adjustment, and a more marked increase in the odds of GAD. A similar pattern was observed in relation to depression. Prevalence of current SUDs remained very low in both groups from adolescence to young adulthood. From the descriptive analysis in the complete cases (online Supplementary Table S5), mental health symptomatology in late adolescence did not appear particularly consistent into young adulthood.

**Table 3.** Proportions and odds ratios of change per year in current mental health outcomes from 18 to 25 years in extremely preterm/extremely low birthweight (EP/ELBW) and normal birthweight (NBW) control groups

	18 years		25 years		Unadjusted			Adjusted	
	EP/ELBW *n* = 215	NBW *n* = 157	EP/ELBW *n* = 174	NBW *n* = 139	Group × age interaction	Age effect		Age effect	
Outcome	*n* (%)	*n* (%)	*n* (%)	*n* (%)	*p*	OR (95% CI)	*p*	aOR (95% CI)	*p*
ADHD symptoms	42 (21%)	15 (10%)	29 (19%)	13 (10%)	0.63	0.99 (0.93–1.06)	0.76	1.00 (0.92–1.07)	0.93
ADHD diagnosis	30 (15%)	11 (7%)	7 (5%)	5 (4%)	0.36	0.87 (0.79–0.95)^a^	0.003^a^	0.87 (0.80–0.95)^a^	0.002^a^
Current anxiety symptoms	34 (18%)	20 (14%)	40 (24%)	29 (21%)	0.75	1.07 (1.01–1.14)	0.03	1.06 (0.98–1.13)	0.14
Current depression symptoms	43 (23%)	32 (22%)	49 (29%)	34 (24%)	0.94	1.06 (0.99–1.14)	0.10	1.05 (0.97–1.14)	0.23
Current anxiety or depression symptoms	53 (28%)	39 (26%)	61 (36%)	42 (30%)	0.66	1.07 (1.00–1.13)	0.05	1.06 (0.99–1.14)	0.10
Generalized anxiety disorder	10 (5%)	6 (4%)	23 (15%)	12 (9%)	0.80	1.22 (1.10–1.35)	<0.001	1.21 (1.09–1.35)	0.001
Major depressive episode	8 (4%)	3 (2%)	19 (12%)	9 (7%)	0.92	1.20 (1.10–1.30)^a^	<0.001^a^	1.33 (1.04–1.70)	0.02
Substance use disorder	5 (2%)	2 (1%)	3 (2%)	2 (2%)	0.55	1.02 (0.86–1.22)	0.80	1.02 (0.89–1.16)^a^	0.80^a^

aOR, adjusted odds ratio; CI, confidence interval.

Denominators vary slightly due to missing data. Adjusted results are adjusted for sex, birthweight s.d. score, lower maternal education, and excludes those with IQs more than 2s.d. below the control group mean.

a Analysis used GEE with exchangeable correlation matrix to account for repeated measures on individuals and robust standard errors due to non-convergence of mixed model.

## Discussion

This study reports encouraging mental health outcomes in a contemporary cohort of adults born EP/ELBW, with similar outcomes for ADHD, anxiety, mood, and substance use to NBW controls at 25 years of age. Although some aspects of mental health changed in prevalence from adolescence to adulthood, the patterns over time were similar between EP/ELBW and NBW young people, indicating that EP/ELBW birth was not a strong risk factor for a poor transition into adulthood in terms of mental health.

At 25 years of age, ADHD prevalence was similar between young people born EP/ELBW and NBW and declined with age from adolescence in both groups. Our EP/ELBW group had lower ADHD prevalence than that reported in IPD meta-analysis (Anderson et al., 2021) and in the Bavarian Longitudinal Study of VLBW young adults using parental report (Breeman et al., 2016), but similar to that reported in another longitudinal study with a relatively modest VLBW sample size (*n* = 44) (Lærum et al., 2017). For both groups, the estimated prevalence of ADHD was broadly similar to the 4.2% prevalence in adults in high-income countries (Fayyad et al., 2007). The small group difference in ADHD symptoms was somewhat surprising given the strong association of preterm birth with childhood attention difficulties, but does align with some reports from pre-1990 cohorts (Boyle et al., 2011; Strang-Karlsson et al., 2008). Our finding of decreasing odds of ADHD over time is in line with the pattern reported in the Bavarian Longitudinal Study (Breeman et al., 2016). However, we also found that the odds of elevated ADHD symptoms remained consistent with age. This may suggest a reduced functional impact or compensation for attention difficulties due to change in contextual factors, such as an increased capacity in adulthood to choose employment and education options that fit with one's skills and weaknesses (Nigg, Sibley, Thapar, & Karalunas, 2020), as opposed to a resolution of attention difficulties by cognitive maturation. Further research is needed to understand the consistency of attention problems at the level of the individual over this important maturational period.

The young adult outcomes in other mental health domains were also generally positive. Although experiences of anxiety and depression were relatively common, the odds of these difficulties did not vary substantially by birth group. This finding stands in contrast to evidence of childhood outcomes, generally reflecting parental reports, and to much of the literature amongst adults born ELBW or VLBW prior to 1990, which highlights modest increases in mental health symptomatology, particularly for internalizing behaviors in adulthood (Boyle et al., 2011; Lærum et al., 2017, 2019; Mathewson et al., 2017; Nosarti et al., 2012). Regarding the associations of preterm birth and disorders reported in IPD meta-analysis (Anderson et al., 2021), we found similar point estimates for the associations of EP/ELBW birth with lifetime anxiety disorders but lower odds for mood disorders, and weaker associations for current symptoms of anxiety and depression. This discrepancy could reflect limited power in the current study, although our sample is of reasonable size in the context of the wider literature and used multiple imputation to minimize the impact of biased attrition. Furthermore, our results are broadly in keeping with those of the Bavarian Longitudinal Study, who found VP/VLBW birth was associated with very weak evidence of increased rates of anxiety and a small increase in risk for mood disorder at 26 years which was attenuated after adjustment for multiple comparisons (Jaekel et al., 2018). Another longitudinal cohort study found increased mood disorders amongst VLBW 26-year-olds compared with controls, but noted an unexpectedly low prevalence of these disorders in their control group (Lærum et al., 2017). The prevalence of lifetime anxiety disorders in our control group (27%) was consistent with that reported from the Australian Bureau of Statistics' 2007 National Survey of Mental Health and Wellbeing (NSMHW) in Australian adults (26%) (Slade, Johnston, Oakley Browne, Andrews, & Whiteford, 2009). However, we did find a higher lifetime prevalence of mood disorder (35% of controls) than in the NSMHW (15%) (Slade et al., 2009). The confidence intervals around the prevalence estimates in the current study were wide, making direct comparisons with community prevalence somewhat challenging. Nevertheless, the rates of anxiety and depression symptoms on questionnaire were broadly similar to recent population-level rates of psychological distress reported on the Kessler Psychological Distress Scale, or K10. For instance, the 2017–18 Australian National Health Survey reported that 37% of people aged 25–34 had moderate or greater psychological distress, and 13% had high or very high levels of distress (Australian Bureau of Statistics, 2018). In the present study, rates of current symptoms in the control group were 32% for depression symptoms and 25% for anxiety symptoms. Our findings of similar outcomes for EP/ELBW and NBW young people may differ from the evidence in childhood as a result of developmental shifts in emotional functioning, and the use of self-report rather than parental ratings (De Los Reyes & Kazdin, 2005). Future studies would benefit from examination of resilience factors to understand heterogeneity in adult mental health outcomes.

In both birth groups, the pattern of change in depression and anxiety prevalence from adolescence to adulthood was similar. More participants reported symptoms consistent with a MDE and GAD in young adulthood than adolescence, although there was less change in the rates of high symptoms of anxiety or depression on questionnaires. Cohorts born before 1990 have had varying findings on this front; Van Lieshout et al. found a gradual increase in internalizing symptoms over time amongst their ELBW cohort compared with a decrease in controls from adolescence to the mid-30s (Van Lieshout et al., 2018), while others have reported that young people born VLBW and full-term experience similarly increasing trajectories of anxiety and mood disorders into their mid-20s (Jaekel et al., 2018; Lærum et al., 2017). It is therefore likely that the change over time found in our study reflects a genuine increase in mood and anxiety difficulties across this important developmental period. It is worth noting that we found limited evidence of individual-level consistency over time in the complete cases. However, this is interpreted with some caution given the potential for bias due to attrition, and further research is needed to better delineate the individual-level trajectories of mental health over time.

Our finding that young people born EP/ELBW were not more vulnerable to SUDs than their NBW peers is consistent with previous studies from pre-1990 cohorts that have indicated VP or EP birth does not elevate the odds of substance use problems and risk-taking behaviors (Lærum et al., 2017; Nosarti et al., 2012). While earlier reports indicated there may be a notable decrease in the risk of current and lifetime substance use problems amongst adults born ELBW (Van Lieshout et al., 2015), the group difference in the present study was much less pronounced. Current SUDs were infrequent in both groups at both time points, and were of lower prevalence than in some other reports from the ELBW population (Van Lieshout et al., 2015). The lifetime prevalence was also lower than the 25% prevalence in Australian population estimated from the NSMHW (Slade et al., 2009). This may reflect a broader social shift in Australia away from risky substance use in youth over recent years (Australian Institute of Health and Welfare, 2020).

The current study reports a long-term follow-up of an extremely well-characterized, geographical cohort of EP/ELBW survivors and matched NBW controls. It used robust clinical measures to examine outcomes of interest dimensionally and categorically. This study was conducted prior to the COVID-19 pandemic and so this was not an interpretive consideration. We also acknowledge some limitations. Like many longitudinal studies, some participants were lost to follow-up. However, by virtue of the longitudinal nature of this cohort, we were able to identify variables collected prior to the 25-year follow-up that were associated with loss to follow-up, which were subsequently used in our multiple imputation procedure. For comparison, we also conducted a complete case analysis, which showed a generally similar pattern of results. We utilized self-report measures, and future studies may benefit from the use of multi-informant approaches to yield more robust data. We did not have measures of parental mental health. Given that preterm birth can affect parents' mental health, this information may provide important context to the psychiatric outcomes of premature birth. It was also beyond the scope of this paper to examine other aspects of mental health within families, such as exploring the association of twin-ship with mental health outcomes. Future studies exploring mental health experiences at the level of the family unit would be a valuable addition to the literature. Differences in healthcare around the world are an additional consideration regarding outcomes of prematurity. This study and much of the relevant existing evidence report on cohorts born in countries and regions with broadly comparable universal public health systems (e.g. Australia, UK, Canada, Western Europe). Interestingly, although there are limited data comparing mental health outcomes between international samples, a previous paper has demonstrated there is consistency in the emotional and behavioral outcomes of children born ELBW in the USA, Canada, the Netherlands, and Germany (Hille et al., 2001). Finally, we acknowledge issues of power, as referenced above. This study was also not powered to examine heterotypic trajectories of specific diagnoses, patterns of comorbidity, or to identify subgroups of individuals with differing courses of mental health. These are important avenues for future research (Copeland, Angold, Shanahan, & Costello, 2014).

In conclusion, this long-term follow-up of one of the earliest geographical cohorts of EP/ELBW infants born in the contemporary era indicated generally positive young adult mental health outcomes compared with NBW peers. However, ADHD, anxiety, depression, and SUDs were still relatively common experiences, and our findings highlight the importance of mental health support for young adults generally.

## Supplementary material

For supplementary material accompanying this paper visit https://doi.org/10.1017/S0033291722002276. click here to view supplementary material

## References

[ref1] Anderson, P. J., de Miranda, D. M., Albuquerque, M. R., Indredavik, M. S., Evensen, K. A. I., Van Lieshout, R., … Doyle, L. W. (2021). Psychiatric disorders in individuals born very preterm/very low-birth weight: An individual participant data (IPD) meta-analysis. EClinicalMedicine, 42, 101216. 10.1016/j.eclinm.2021.101216. 34901794 PMC8639417

[ref2] Anderson, P. J., & Doyle, L. W. (2003). Neurobehavioural outcomes of school-age children born extremely low birth weight or very preterm in the 1990s. Jama, 289(24), 3264–3272. 12824207 10.1001/jama.289.24.3264

[ref3] Australian Institute of Health and Welfare. (2020). National drug strategy household survey *2019*. Canberra: AIHW. Retrieved from https://www.aihw.gov.au/reports/illicit-use-of-drugs/national-drug-strategy-household-survey-2019.

[ref4] Bachmann, C. S., Risnes, K., Bjørngaard, J. H., Schei, J., & Pape, K. (2021). Association of preterm birth with prescription of psychotropic drugs in adolescence and young adulthood. JAMA Network Open, 4(3), e211420. doi: 10.1001/jamanetworkopen.2021.1420 33710290 PMC7955275

[ref5] Beck, A. T., & Steer, R. A. (1990). Beck anxiety inventory manual. San Antonio, TX: Psychological Corporation.

[ref6] Boyle, M. H., Miskovic, V., Van Lieshout, R., Duncan, L., Schmidt, L. A., Hoult, L., … Saigal, S. (2011). Psychopathology in young adults born extremely low birth weight. Psychological Medicine, 41, 1763–1774. 21134317 10.1017/S0033291710002357

[ref7] Breeman, L. D., Jaekel, J., Baumann, N., Bartmann, P., & Wolke, D. (2016). Attention problems in very preterm children from childhood to adulthood: The Bavarian longitudinal study. Journal of Child Psychology and Psychiatry, 57(2), 132–140. doi: 10.1111/jcpp.12456 26287264

[ref8] Burnett, A., Davey, C. G., Wood, S. J., Wilson-Ching, M., Molloy, C., Cheong, J. L., … Anderson, P. J. (2014). Extremely preterm birth and adolescent mental health in a geographical cohort born in the 1990s. Psychological Medicine, 44(7), 1533–1544. 23981686 10.1017/S0033291713002158

[ref9] Carlin, J. B., Gurrin, L. C., Sterne, J. A. C., Morley, R., & Dwyer, T. (2005). Regression models for twin studies: A critical review. International Journal of Epidemiology, 34(5), 1089–1099. 16087687 10.1093/ije/dyi153

[ref10] Caspi, A., Houts, R. M., Ambler, A., Danese, A., Elliott, M. L., Hariri, A., … Moffitt, T. E. (2020). Longitudinal assessment of mental health disorders and comorbidities across 4 decades among participants in the Dunedin birth cohort study. JAMA Network Open, 3(4), e203221–e203221. doi: 10.1001/jamanetworkopen.2020.3221 32315069 PMC7175086

[ref11] Cheong, J. L. Y., Wark, J. D., Cheung, M. M., Irving, L., Burnett, A. C., Lee, K. J., … Doyle, L. W. (2019). Impact of extreme prematurity or extreme low birth weight on young adult health and well-being: the Victorian infant collaborative study (VICS) 1991–1992 longitudinal cohort study protocol. BMJ Open, 9(5), e030345. doi: 10.1136/bmjopen-2019-030345 PMC6527969 31072865

[ref12] Copeland, W. E., Angold, A., Shanahan, L., & Costello, E. J. (2014). Longitudinal patterns of anxiety from childhood to adulthood: The Great Smoky Mountains Study. Journal of the American Academy of Child & Adolescent Psychiatry, 53(1), 21–33. doi: 10.1016/j.jaac.2013.09.017 24342383 PMC3939681

[ref13] De Los Reyes, A., & Kazdin, A. E. (2005). Informant discrepancies in the assessment of childhood psychopathology: A critical review, theoretical framework, and recommendations for further study. Psychological Bulletin, 131(4), 483–509. 16060799 10.1037/0033-2909.131.4.483

[ref14] Doyle, L. W. (2001). Outcome at 5 years of age of children 23–27 weeks’ gestation: Refining the prognosis. Pediatrics, 108(1), 134–141. 11433066 10.1542/peds.108.1.134

[ref15] Doyle, L. W., & Victorian Infant Collaborative Study Group. (2004). Evaluation of neonatal intensive care for extremely low birth weight infants in Victoria over two decades: I. Effectiveness. Pediatrics, 113(3), 505–509. 14993541 10.1542/peds.113.3.505

[ref16] Eaton, W. W., Muntaner, C., Smith, C., Tien, A., & Ybarra, M. (2004). Center for epidemiologic studies depression scale: Review and revision (CESD and CESD-R). In M. E. Maruish (Ed.), The use of psychological testing for treatment planning and outcomes assessment (3rd ed., pp. 363–377). Mahwah, NJ: Lawrence Erlbaum.

[ref17] Fayyad, J., De Graaf, R., Kessler, R., Alonso, J., Angermeyer, M., Demyttenaere, K., … Jin, R. (2007). Cross-national prevalence and correlates of adult attention-deficit hyperactivity disorder. British Journal of Psychiatry, 190, 402–409. doi: 10.1192/bjp.bp.106.034389 17470954

[ref18] First, M. B., Spitzer, R. L., Gibbon, M., & Williams, J. B. W. (2002). Structured clinical interview for DSM-IV-TR axis I disorders, research version, non-patient edition *(*SCID-I/NP*)*. New York: Biometrics Research, New York State Psychiatric Institute.

[ref19] Hille, E. T. M., den Ouden, A. L., Saigal, S., Wolke, D., Lambert, M., Whitaker, A., … Paneth, N. (2001). Behavioural problems in children who weigh 1000 g or less at birth in four countries. Lancet, 357(9269), 1641–1643. 11425366 10.1016/S0140-6736(00)04818-2

[ref20] Jaekel, J., Baumann, N., Bartmann, P., & Wolke, D. (2018). Mood and anxiety disorders in very preterm/very low-birth weight individuals from 6 to 26 years. Journal of Child Psychology and Psychiatry, 59(1), 88–95. doi: 10.1111/jcpp.12787 28748557

[ref21] Johnson, S., & Marlow, N. (2014). Growing up after extremely preterm birth: Lifespan mental health outcomes. Seminars in Fetal & Neonatal Medicine, 19(2), 97–104. doi: 10.1016/j.siny.2013.11.004 24290907

[ref22] Kessler, R. C., Adler, L., Ames, M., Demler, O., Faraone, S., Hiripi, E., … Walters, E. E. (2005). The world health organization adult ADHD self-report scale (ASRS): A short screening scale for use in the general population. Psychological Medicine, 35(2), 245–256. doi: 10.1017/s0033291704002892 15841682

[ref23] Lærum, A. M. W., Reitan, S. K., Evensen, K. A. I., Lydersen, S., Brubakk, A.-M., Skranes, J., & Indredavik, M. S. (2017). Psychiatric disorders and general functioning in low birth weight adults: A longitudinal study. Pediatrics, 139(2), e20162135. doi: 10.1542/peds.2016-2135 28123043

[ref24] Lærum, A. M. W., Reitan, S. K., Evensen, K. A. I., Lydersen, S., Brubakk, A. M., Skranes, J., & Indredavik, M. S. (2019). Psychiatric symptoms and risk factors in adults born preterm with very low birthweight or born small for gestational age at term. BMC Psychiatry, 19(1), 223. doi: 10.1186/s12888-019-2202-8 31315591 PMC6636134

[ref25] Mathewson, K. J., Chow, C. H., Dobson, K. G., Pope, E. I., Schmidt, L. A., & Van Lieshout, R. J. (2017). Mental health of extremely low birth weight survivors: A systematic review and meta-analysis. Psychological Bulletin, 143(4), 347–383. doi: 10.1037/bul0000091 28191983

[ref26] Nigg, J. T., Sibley, M. H., Thapar, A., & Karalunas, S. L. (2020). Development of ADHD: Etiology, heterogeneity, and early life course. Annual Review of Developmental Psychology, 2(1), 559–583. doi: 10.1146/annurev-devpsych-060320-093413 PMC8336725 34368774

[ref27] Nosarti, C., Reichenberg, A., Murray, R. M., Cnattingius, S., Lambe, M. P., Yin, L., … Hultman, C. M. (2012). Preterm birth and psychiatric disorders in young adult life. Archives of General Psychiatry, 69(6), 610–617. 10.1001/archgenpsychiatry.2011.1374 22660967

[ref28] Robinson, R., Lahti-Pulkkinen, M., Schnitzlein, D., Voit, F., Girchenko, P., Wolke, D., … Räikkönen, K. (2020). Mental health outcomes of adults born very preterm or with very low birth weight: A systematic review. Seminars in Fetal & Neonatal Medicine, 25(3), 101113. doi: 10.1016/j.siny.2020.101113. 32402835

[ref29] Rubin, D. (1987). Multiple imputation for nonresponse in surveys. New York: John Wiley & Sons Inc.

[ref30] Slade, T., Johnston, A., Oakley Browne, M. A., Andrews, G., & Whiteford, H. (2009). 2007 National survey of mental health and wellbeing: Methods and key findings. Australian and New Zealand Journal of Psychiatry, 43(7), 594–605. doi: 10.1080/00048670902970882 19530016

[ref31] StataCorp (2019). Stata statistical software: Release 16. College Station, TX: StataCorp LLC.

[ref32] Strang-Karlsson, S., Räikkönen, K., Pesonen, A.-K., Kajantie, E., Paavonen, E. J., Lahti, J., … Andersson, S. (2008). Very low birth weight and behavioral symptoms of attention deficit hyperactivity disorder in young adulthood: The Helsinki study of very-low-birth-weight adults. American Journal of Psychiatry, 165(10), 1345–1353. 18628349 10.1176/appi.ajp.2008.08010085

[ref33] Van Lieshout, R. J., Boyle, M. H., Saigal, S., Morrison, K., & Schmidt, L. A. (2015). Mental health of extremely low birth weight survivors in their 30s. Pediatrics, 135(3), 452–459. doi: 10.1542/peds.2014-3143 25667243

[ref34] Van Lieshout, R. J., Ferro, M. A., Schmidt, L. A., Boyle, M. H., Saigal, S., Morrison, K. M., & Mathewson, K. J. (2018). Trajectories of psychopathology in extremely low birth weight survivors from early adolescence to adulthood: A 20-year longitudinal study. Journal of Child Psychology and Psychiatry, 59(11), 1192–1200. doi: 10.1111/jcpp.12909 29667718 PMC6193866

[ref35] Victorian Infant Collaborative Study Group. (1997). Outcome at 2 years of children 23–27 weeks’ gestation born in Victoria in 1991–92. Journal of Paediatrics and Child Health, 33(2), 161–165. 9145362

[ref36] Weller, E. B., Weller, R. A., Rooney, M. T., & Fristad, M. A. (1999). Children's interview for psychiatric syndromes *(*ChIPS*)*. Washington, DC: American Psychiatric Press. 10.1097/00004583-200001000-00019 10638070

