# Supplementary material for: Mental health in young adults born extremely preterm or extremely low birthweight with contemporary neonatal intensive care
Source: Psychol Med. 2022 Jul 22;53(11):5227–34. doi: 10.1017/S0033291722002276 (PMC10476050; doi:10.1017/S0033291722002276)
Supplement: Supplementary file 1 [file S0033291722002276sup001.docx]

**Supporting Table 1.** **Distribution of Beck Anxiety Inventory and Center for Epidemiologic Studies Depression Scale Revised total scores at age 25 years by birth-group.**

|  | EP/ELBW | NBW | Wilcoxon rank-sum *p* |
| --- | --- | --- | --- |
| CESD-R mean (SD) | 12.5 (13.7) | 11.3 (13.6) | - |
| CESD-R median [IQR] | 8 [2, 18] | 6 [2, 15] | 0.43 |
| BAI mean (SD) | 9.9 (10.5) | 9.1 (9.9) | - |
| BAI median [IQR] | 6 [2, 14] | 5.25 [2, 12.6] | 0.80 |

BAI: Beck Anxiety Inventory; CESD-R: Center for Epidemiologic Studies Depression Scale Revised; EP/ELBW: extremely preterm/extremely low birthweight; IQR: interquartile range; NBW: normal birthweight; SD: standard deviation.

**Supporting Table 2. DSM-IV Disorders Assessed at 25 years.**

| **Anxiety Disorders** | **Mood disorders** | **Substance use disorders** |
| --- | --- | --- |
| - Panic disorder - Agoraphobia - Social phobia - Specific phobia - Obsessive compulsive disorder - Post-traumatic stress disorder - Generalised anxiety disorder^ - Anxiety disorder due to a general medical condition - Substance-induced anxiety disorder - Anxiety disorder not otherwise specified | - Major depressive disorder with current/past major depressive episode - Bipolar disorder - Dysthymic disorder^ - Mood disorder due to a general medical condition - Substance-induced mood disorder - Mood disorder not otherwise specified | - Current alcohol abuse or dependence - Past alcohol use abuse or dependence - Current non-alcohol abuse or dependence - Past non-alcohol abuse or dependence |

^Current disorder only; due to low numbers, alcohol and non-alcohol substance abuse and dependence were combined to create variables reflecting current/past substance use disorder.

**Supporting Table 3.** **Additional details of multiple imputation analysis**.

Multiple imputation was performed separately for each outcome using “mi impute chained” in Stata v16.1. All univariate imputation models included the outcome at age 25 and 18 years, the covariates sex (male/female), birthweight SD score, maternal education (lower/higher), and the auxiliary variables provided in the table below. Auxiliary variables were selected from follow-up at 8, 18, and 25 years. Imputations were performed by birth group (except for ADHD diagnosis, where the groups were combined due to low cell counts).

A list of auxiliary variables was originally identified based on substantive knowledge. For each outcome, auxiliary variables that had moderate to large correlations (>0.2) with the outcome at 25 or 18 years or were strong predictors of missingness in the outcome at 25 or 18 years were identified from this list and included in the imputation procedure for that outcome. Problematic auxiliary variables (those that caused the imputation procedure to fail) were subsequently identified and removed. The table below provides details of the auxiliary variables included in the imputation process and summary statistics describing the relationship between the auxiliary variables and the incomplete outcome at age 18 and 25 years.

| **Outcome** | **Auxiliary variable** | **Correlation with outcome at 25 years** | **Correlation with outcome at 18 years** | **Odds ratio for missingness in outcome at 25 years (95% CI)** | **Odds ratio for missingness in outcome at 18 years (95% CI)** |
| --- | --- | --- | --- | --- | --- |
| ADHD diagnosis | Moderate or severe disability (8 y) | 0.03 | 0.17 | 4.73 (2.54, 8.81) | 3.38 (1.96, 5.81) |
|  | Full-scale IQ (8 y) | 0.06 | -0.23 | 0.97 (0.96, 0.98) | 0.97 (0.96, 0.98) |
|  | Full-scale IQ (18 y) | 0.04 | -0.24 | 0.97 (0.95, 0.98) | 0.92 (0.89, 0.95) |
|  | BAI score (18 y) | 0.12 | 0.35 | 1.00 (0.97, 1.02) | 0.99 (0.92, 1.06) |
|  | CESD-R score (18 y) | 0.10 | 0.27 | 1.00 (0.98, 1.02) | 1.02 (0.99, 1.06) |
|  | BASC Attention Problems T-score (8 y) | 0.00 | 0.28 | 1.01 (0.99, 1.03) | 1.02 (1.00, 1.04) |
|  | BASC Hyperactivity T-score (8 y) | 0.13 | 0.25 | 1.02 (1.00, 1.03) | 1.02 (1.00, 1.03) |
|  | BASC Externalizing Problems T-score (8 y) | 0.14 | 0.29 | 1.03 (1.01, 1.04) | 1.03 (1.01, 1.04) |
|  | BASC Behavioral Symptoms Index T-score (8 y) | 0.11 | 0.25 | 1.02 (1.00, 1.03) | 1.03 (1.01, 1.05) |
|  | BRIEF Behavioral Regulation Index T-score (8 y) | 0.15 | 0.22 | 1.02 (1.01, 1.04) | 1.04 (1.02, 1.06) |
|  | BRIEF Metacognition Index T-score (8 y) | 0.02 | 0.22 | 1.02 (1.00, 1.04) | 1.03 (1.01, 1.06) |
|  | BRIEF Global Executive Composite T-score (8 y) | 0.08 | 0.23 | 1.02 (1.00, 1.04) | 1.04 (1.02, 1.06) |
| ADHD symptoms | BAI score (18 y) | 0.18 | 0.36 | 0.99 (0.97, 1.02) | 1.00 (0.94, 1.06) |
|  | CESD-R score (18 y) | 0.24 | 0.32 | 0.99 (0.97, 1.01) | 1.03 (0.99, 1.06) |
|  | Full-scale IQ (18 y) | 0.04 | -0.2 | 0.97 (0.95, 0.98) | 0.93 (0.91, 0.96) |
|  | BAI score (25 y) | 0.39 | 0.1 | 1.04 (1.00, 1.08) | 1.02 (0.99, 1.05) |
|  | CESD-R score (25 y) | 0.38 | 0.15 | 1.00 (0.97, 1.03) | 1.01 (0.99, 1.03) |
| High BAI scores | Full-scale IQ (18 y) | -0.13 | -0.04 | 0.96 (0.95, 0.98) | 0.95 (0.93, 0.97) |
|  | CESD-R score (18 y) | 0.22 | 0.54 | 0.99 (0.97, 1.02) | 0.91 (0.70, 1.19) |
|  | CESD-R score (25 y) | 0.67 | 0.29 | 1.01 (0.91, 1.13) | 1.02 (1.00, 1.04) |
| Any anxiety disorder | Full-scale IQ (18 y) | -0.02 | -0.03 | 0.97 (0.96, 0.98) | 0.92 (0.90, 0.95) |
|  | BAI score (18 y) | 0.24 | 0.37 | 0.99 (0.97, 1.02) | 0.99 (0.91, 1.07) |
|  | CESD-R score (18 y) | 0.22 | 0.34 | 0.99 (0.97, 1.01) | 0.98 (0.91, 1.05) |
|  | BAI score (25 y) | 0.44 | 0.23 | 1.03 (0.99, 1.08) | 1.02 (0.99, 1.05) |
|  | CESD-R score (25 y) | 0.41 | 0.24 | 1.00 (0.97, 1.03) | 1.01 (0.99, 1.03) |
| High CESD-R scores | Full-scale IQ (18 y) | -0.14 | 0.04 | 0.96 (0.95, 0.98) | 0.94 (0.92, 0.96) |
|  | BAI score (18 y) | 0.36 | 0.52 | 0.99 (0.96, 1.01) | 1.05 (0.96, 1.15) |
|  | BASC Depression T-score (8 y) | 0.21 | 0.17 | 1.02 (1.00, 1.03) | 1.03 (1.01, 1.05) |
|  | BRIEF Global Executive Composite T-score (8 y) | 0.21 | 0.08 | 1.02 (1.01, 1.04) | 1.04 (1.02, 1.06) |
|  | BAI score (25 y) | 0.58 | 0.3 | No cases observed when outcome is missing | 1.03 (1.00, 1.06) |
| Any mood disorder | Full-scale IQ (18 y) | -0.03 | -0.06 | 0.97 (0.96, 0.98) | 0.92 (0.90, 0.95) |
|  | BAI score (18 y) | 0.31 | 0.43 | 0.99 (0.97, 1.02) | 0.99 (0.91, 1.07) |
|  | CESD-R score (18 y) | 0.33 | 0.51 | 0.99 (0.97, 1.01) | 0.98 (0.91, 1.05) |
|  | BAI score (25 y) | 0.47 | 0.3 | 1.03 (0.99, 1.08) | 1.02 (0.99, 1.05) |
|  | CESD-R score (25 y) | 0.58 | 0.38 | 1.00 (0.97, 1.03) | 1.01 (0.99, 1.03) |
| Any substance use disorder | BAI score (18 y) | 0.30 | 0.19 | 0.99 (0.97, 1.02) | 1.00 (0.93, 1.07) |
|  | BAI score (25 y) | 0.06 | -0.04 | 1.03 (0.99, 1.08) | 1.02 (0.99, 1.05) |
|  | CESD-R score (25 y) | 0.14 | 0.05 | 1.00 (0.97, 1.03) | 1.01 (0.99, 1.03) |
| Any mood or anxiety disorder | Full-scale IQ (8 y) | -0.05 | -0.02 | 0.97 (0.96, 0.99) | 0.97 (0.96, 0.98) |
|  | Full-scale IQ (18 y) | -0.02 | -0.05 | 0.97 (0.96, 0.98) | 0.92 (0.90, 0.95) |
|  | BAI score (18 y) | 0.33 | 0.4 | 0.99 (0.97, 1.02) | 0.99 (0.91, 1.07) |
|  | CESD-R score (18 y) | 0.36 | 0.46 | 0.99 (0.97, 1.01) | 0.98 (0.91, 1.05) |
|  | BAI score (25 y) | 0.50 | 0.3 | 1.03 (0.99, 1.08) | 1.02 (0.99, 1.05) |
|  | CESD-R score (25 y) | 0.56 | 0.34 | 1.00 (0.97, 1.03) | 1.01 (0.99, 1.03) |
|  | BASC Depression T-score (8 y) | 0.24 | 0.15 | 1.02 (1.00, 1.03) | 1.03 (1.01, 1.04) |
| Any depression or anxiety symptoms | BAI score (18 y) | 0.34 | 0.7 | 0.99 (0.96, 1.02) | 0.98 (0.83, 1.17) |
|  | CESD-R score (18 y) | 0.39 | 0.73 | 0.99 (0.97, 1.02) | No cases observed when outcome is missing |
|  | CESD-R score (25 y) | 0.76 | 0.42 | 1.01 (0.91, 1.13) | 1.02 (1.00, 1.04) |
|  | BRIEF Global Executive Composite T-score (8 y) | 0.21 | 0.12 | 1.03 (1.01, 1.05) | 1.04 (1.02, 1.06) |

Note: correlations and odds ratios are calculated using data from the available cases. BAI: Beck Anxiety Inventory; BASC: Behavior Assessment System for Children; BRIEF: Behavior Rating Inventory of Executive Function; CESD-R: Center for Epidemiologic Studies Depression Scale Revised.

**Supporting Table 4.** Complete case analysis of mental health outcomes at 25 years in extremely preterm/extremely low birthweight (EP/ELBW) and normal birthweight (NBW) control groups

|  | EP/ELBW n (%) | NBW n (%) | *Unadjusted odds ratio (95% CI)* | *p* | *Adjusted odds ratio (95% CI)* | *Adj. p* |
| --- | --- | --- | --- | --- | --- | --- |
| ADHD symptoms | 32/162 (20%) | 13/131 (10%) | 2.05 (1.02, 4.13) | 0.04 | 1.94 (0.82, 4.57) | 0.13 |
| ADHD diagnosis | 8/160 (5%) | 5/131 (4%) | 1.20 (0.37, 3.88) | 0.76 | 1.70 (0.44, 6.56) | 0.44 |
| Current anxiety symptoms^#^ | 40/169 (24%) | 29/139 (21%) | 1.21 (0.71, 2.05) | 0.49 | 1.30 (0.65, 2.58) | 0.45 |
| Current depression symptoms^ | 49/170 (29%) | 34/139 (24%) | 1.24 (0.75, 2.07) | 0.40 | 0.81 (0.41, 1.60) | 0.55 |
| Current anxiety or mood symptoms^#^^ | 61 (36%) | 42 (30%) | 1.31 (0.82, 2.11) | 0.26 | 1.05 (0.58, 1.90) | 0.88 |
| Any anxiety disorder | 53/158 (34%) | 34/130 (26%) | 1.46 (0.87, 2.44) | 0.15 | 1.66 (0.87, 3.16) | 0.12 |
| Any mood disorder | 58/158 (37%) | 39/130 (30%) | 1.36 (0.82, 2.23) | 0.23 | 0.96 (0.50, 1.86) | 0.91 |
| Any substance-use disorder | 20/158 (13%) | 14/130 (11%) | 1.15 (0.55, 2.40) | 0.70 | 1.01 (0.35, 2.88)^$^ | 0.98 |

^#^on Beck Anxiety Inventory; ^on Center for Epidemiologic Studies Depression Scale-Revised; Disorders include current and lifetime; Adjusted results are adjusted for sex, birthweight SD score, and excludes those with IQs more than 2SD below the control group mean at 18 years or at a previous follow-up if 18 year data were unavailable. ^$^Results from logistic regression model with cluster-robust standard errors due to failure when fitting model using GEEs; CI = confidence interval

**Supporting Table 5.** Complete case analysis of change over time in current mental health outcomes from 18 to 25 years in young people born EP/ELBW and NBW.

|  |  | **EP/ELBW** | | **NBW** | |
| --- | --- | --- | --- | --- | --- |
|  |  | **Diagnosis at 25** | | **Diagnosis at 25** | |
|  |  | **Yes** | **No** | **Yes** | **No** |
| **ADHD symptoms at 18** | **Yes** | 7 | 25 | 4 | 4 |
|  | **No** | 18 | 90 | 6 | 92 |
| **ADHD at 18** | **Yes** | 2 | 21 | 0 | 4 |
|  | **No** | 5 | 109 | 5 | 99 |
| **Current anxiety symptoms**^#^ | **Yes** | 12 | 14 | 5 | 11 |
|  | **No** | 18 | 97 | 16 | 81 |
| **Current depression symptoms^** | **Yes** | 19 | 14 | 14 | 7 |
|  | **No** | 16 | 91 | 13 | 79 |
| **Any anxiety or mood symptoms** | **Yes** | 23 | 17 | 18 | 9 |
|  | **No** | 23 | 77 | 16 | 70 |
| **Generalized anxiety disorder** | **Yes** | 3 | 6 | 2 | 2 |
|  | **No** | 16 | 116 | 9 | 95 |
| **Current major depressive episode** | **Yes** | 4 | 2 | 1 | 0 |
|  | **No** | 15 | 120 | 7 | 100 |
| **Any current substance-use disorder** | **Yes** | 1 | 3 | 0 | 1 |
|  | **No** | 2 | 135 | 1 | 106 |

^#^on Beck Anxiety Inventory; ^on Center for Epidemiologic Studies Depression Scale-Revised
